# Mechanism of the reduced shock sensitivity of CL-20/MTNP co-crystals from reactive molecular dynamics simulations

**DOI:** 10.1039/d6ra01177b

**Published:** 2026-04-13

**Authors:** Fuping Wang, Guangyan Du, Huihan Zhang, Liufei Yang, Wanru Bi, Yiyang Li, Yaowen Liu, Wenfang Hou, Songen Wang, Dongqiang Zhang

**Affiliations:** a School of Chemistry and Materials Science, Langfang Normal University Langfang 065000 China wangfuping@lfnu.edu.cn

## Abstract

Elucidating the reasons for the reduced shock sensitivity of 2,4,6,8,10,12-hexanitro-2,4,6,8,10,12-hexaazaisowurtzitane/1-methyl-3,4,5-trinitropyrazole (CL-20/MTNP) compared with CL-20 is crucial for understanding the shock initiation of CL-20/MTNP co-crystalline explosives. In this study, molecular dynamics based on the ReaxFF-lg reactive force field was employed to simulate the propagation of the shock wavefront in the CL-20/MTNP co-crystal and corresponding single crystals. Thermodynamic properties and initial chemical reactions were analyzed using in-house programs. The results indicated that the pressure and temperature at the shock wavefront in the CL-20/MTNP co-crystal were not significantly different from those in the CL-20 crystal. During low- and medium-velocity impacts, the reactant molecules of the CL-20/MTNP co-crystal decayed slower than those of the corresponding single crystals. However, during high-velocity impacts, the decay rates of reactant molecules in the three crystals were similar. After the shock wavefront passed through, multiple CL-20 molecules of the CL-20 crystal simultaneously generated a large amount of NO_2_. In the co-crystal, polymerization occurred primarily between CL-20 and MTNP molecules, which likely suppressed polymerization between CL-20 molecules, leading to the prolonged presence of NO_2_ and extended generation of the intermediate product, which delayed the appearance of N_2_. This may be the reason for the reduced impact sensitivity of CL-20/MTNP co-crystalline explosives.

## Abstract 2

For the first time, reactive nonequilibrium molecular dynamics was used to study the mechanism of reduced shock sensitivity of CL-20/MTNP cocrystal.

## Graphical abstract

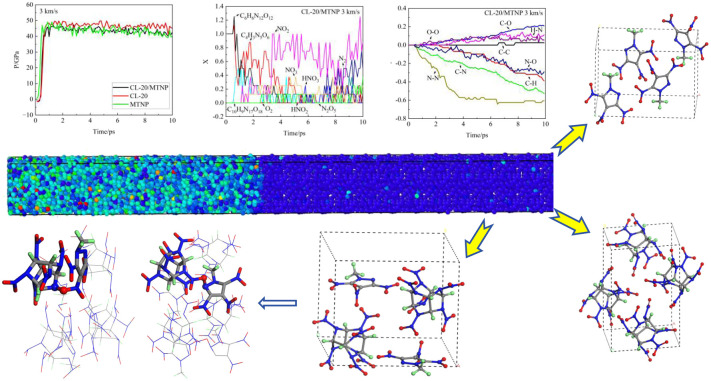

## 1. Introduction

Energetic materials (such as explosives, gunpowder, and propellants) play a crucial role in the military.^1^ Following trinitrotoluene (TNT), cyclotrimethylene trinitramine (RDX), and cyclotetramethylene tetranitramine (HMX), 2,4,6,8,10,12-hexanitro-2,4,6,8,10,12-hexaazaisowurtzitane (CL-20) is a highly promising nitramine explosive with extensive applications.^2^ Owing to its high energy density, CL-20 is extremely sensitive to external stimuli, which is not conducive to its transportation and storage.^3^ Bolton *et al.*^4,5^ first synthesized CL-20/TNT and CL-20/HMX co-crystalline explosives to significantly reduce the impact sensitivity of CL-20. In recent years, researchers have successfully developed a large number of CL-20 co-crystalline explosives to obtain high-energy, low-sensitivity energetic materials.^6^

Considerable progress has been made in understanding the sensitivity of energetic co-crystals from both experimental and theoretical perspectives.^7^ Important global models have revealed that the sensitivity of energetic co-crystals generally follows an intermediate trend between that of their coformers, which can be rationalized by energy density and the propagation of decomposition reactions.^8^ Notably, some earlier reports of exceptions to this trend have been revised by subsequent experimental measurements.^9^ While these researches offer valuable macroscopic trends, the atomistic mechanisms responsible for their reduced sensitivity remain to be fully elucidated, especially early-stage physical responses and bond-breaking governing initiation. Experimental characterization is challenging due to ultra-short initiation time/length scales and CL-20 co-crystals still being in laboratory synthesis. Thus, molecular dynamics simulations offer a powerful means to probe the detailed molecular processes that are difficult to characterize experimentally.^10–12^

Extensive studies using molecular dynamics simulations have shown that the main reasons for the reduced sensitivity of CL-20/HMX, CL-20/TNT, and CL-20/BTF co-crystals are the enhanced intermolecular interactions, reduced free space, and changed environment of CL-20 molecules.^13^ Moreover, Xue *et al.*^14^ confirmed that the main factors contributing to the improved stability of CL-20/HMX co-crystals are molecular stability and intermolecular interactions. Under compressive shear, the sensitivity of CL-20/TNT co-crystals is influenced by the steric hindrance of the constituent molecules.^15^ In contrast, under high-temperature thermal effects, the sensitivity of CL-20/TNT co-crystals is predominantly governed by the carbon-rich clusters generated by the decomposition of TNT.^16^ Similarly, under high-temperature thermal effects, the reduced sensitivity of CL-20/DNB co-crystals is attributed to the generation of a large number of DNB molecules during the initial stage of the chemical reaction, which hinders effective collisions between CL-20 and its intermediate products, thereby reducing the concentration of intermediate products from the thermal decomposition of CL-20.^17^ The decelerated high-temperature thermal decomposition of CL-20/HMX and CL-20/TNT co-crystals is related to changes in the decomposition kinetics of CL-20.^18,19^ Upon impact, the heat released from the decomposition of CL-20 molecules in CL-20/TNT co-crystals accelerates the decomposition of TNT, leading to a decrease in the decomposition rate of CL-20.^20^ In summary, the factors decreasing the sensitivity of co-crystals are complex and multifaceted and vary depending on the loading method and type of co-crystals. Current research on the aftermath of impact is primarily focused on the stable detonation phase, which has a constant wavefront. Consequently, it remains unclear how the chemical reactions in co-crystalline explosives lead to sensitivity reduction in the shock initiation phase, where the shock wave has just entered the crystal and has not yet effectuated stable detonation.

In this study, molecular dynamics simulations were employed to investigate changes in the density, temperature, and pressure of CL-20/MTNP co-crystals and corresponding single crystals induced by the propagation of wavefronts generated by impacts with different velocities. Molecular structure changes and initial chemical reactions were analyzed to explore the underlying reasons for the decrease in sensitivity of CL20/MTNP co-crystalline explosives. This is beneficial for gaining a deeper understanding of the shock initiation of CL-20 co-crystalline explosives, providing guidance and assistance for the design and synthesis of novel CL-20 co-crystalline energetic materials.

## 2. Methods

### 2.1. Construction of unit cells

**Fig. 1:**
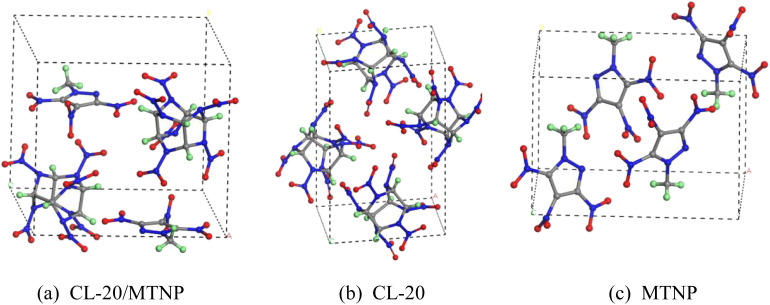
Unit-cell structures of (a) CL-20/MTNP, (b) CL-20, and (c) MTNP crystals. C, H, N, and O atoms are represented by gray, green, blue, and red, respectively. These colors also apply to subsequent figures.

The unit-cell structures of CL-20/MTNP, MTNP, and CL-20 crystals were obtained from experimental results,^21,22^ as shown in Fig. 1. The unit cell of the CL-20/MTNP co-crystal contained two CL-20 molecules (C_6_H_6_N_12_O_12_) and two MTNP molecules (C_4_H_3_N_5_O_6_), in a molar ratio of 1 : 1. Both the CL-20 and MTNP unit cells contained four molecules.

### 2.2. Molecular dynamics simulations

Geometric optimization was performed using the conjugate gradient method and ReaxFF-lg reactive force field in LAMMPS. The system was relaxed for 10 ps at 298 K using the canonical ensemble (NVT). The equilibrium structure at room temperature and pressure was obtained by relaxing the system for 15 ps at 0 GPa and 298 K using the isothermal–isobaric ensemble (*NPT*). To simulate the normal impact state on a plane, the equilibrium structures of the three types of unit cells were extended along the *b*-axis to construct a supercell consisting of tens of thousands of atoms. The boundary condition of the (010) crystal plane containing the origin of the *b*-axis of the supercell was modified to construct a reflecting wall. The length of each unit cell along the *b*-direction was set to one molecular layer. Along the *y*-direction, all atoms were primed to impact the reflecting wall by adding translational velocities of 2, 3, and 4 km s^−1^. The boundary condition of the reflecting wall enabled simulation of the shock wavefront. Propagation was in the opposite direction. The entire impact process was simulated using the microcanonical ensemble (*NVE*), and the calculation was terminated when the shock wavefront reached the end of the crystal. The temperature, pressure, and density of each molecular layer during the propagation of the shock wavefront were calculated. The ReaxFF-lg reactive force field was employed to identify the chemical species and the atoms connected by chemical bonds.

### 2.3. Processing of simulation data

A large number of Python scripts were written to process the data generated by the molecular dynamics simulations.

## 3. Results and discussion

### 3.1. Verification of the applicability of the reactive force field

Table 1 presents the calculated lattice parameters and densities of the three crystals, along with their experimental values. The calculated values were consistent with the experimental values, verifying the applicability of the ReaxFF-lg reactive force field for molecular dynamics simulations of the three crystals. Employing a verified force field ensures that the computational data are accurate and the research results are reliable.

**Table 1:** Unit-cell parameters and densities of CL-20/MTNP, CL-20, and MTNP crystals

Crystals	Methods	*a*/Å	*b*/Å	*c*/Å	Density/g cm^−3^
CL-20/MTNP	Experiments	8.352	11.430	11.804	1.932
	ReaxFF-lg calculations	8.438	11.548	12.064	1.873
	Error/%	1.03	1.03	2.203	−3.05
CL-20	Experiments	8.863	12.593	13.395	2.035
	ReaxFF-lg calculations	9.038	12.842	13.660	1.919
	Error/%	1.97	1.98	1.985	−5.7
MTNP	Experiments	11.921	8.339	8.476	1.711
	ReaxFF-lg calculations	11.621	8.129	8.263	1.847
	Error/%	−2.52	−2.52	−2.51	7.95

### 3.2. Thermodynamic properties

**Fig. 2:**
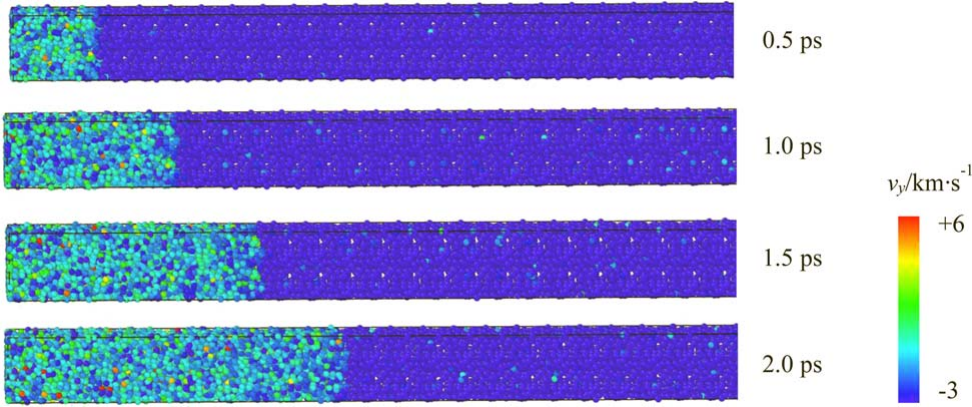
Velocity distribution of atoms in the CL-20/MTNP supercell along the *y*-direction at an impact velocity of 3 km s^−1^.

The velocity distribution of atoms in the supercell near the wall along the *y*-direction at different time points under an impact velocity of 3 km s^−1^ was selected to visually depict the propagation of the shock wave in the CL-20/MTNP co-crystal caused by molecules impacting the reflecting wall, as shown in Fig. 2. The velocity, represented by different colors, ranged from −3 km s^−1^ to 6 km s^−1^. Because all atoms were moving at an initial velocity of −3 km s^−1^, they were colored in blue. When atoms move at a velocity of −*vy* and hit the wall, the velocity of atoms near the wall changes instantaneously, and this change propagates layer by layer to the right, forming a shock wave.

**Fig. 3:**
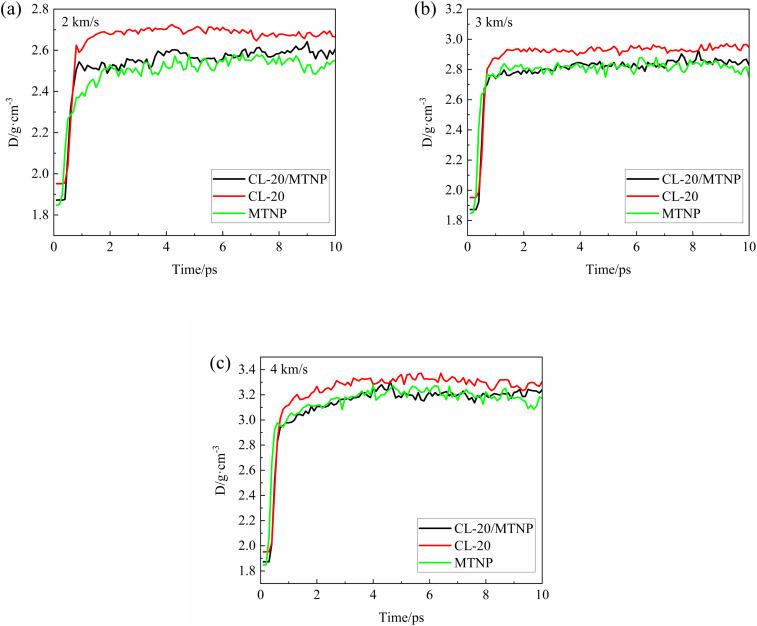
Temporal variation of the densities of CL-20/MTNP, CL-20, and MTNP crystals at different impact velocities: (a) 2 km s^−1^; (b) 3 km s^−1^; (c) 4 km s^−1^.

To investigate the physical response of the crystals after the shock wavefront passed through, thermodynamic properties such as density, pressure, and temperature were tracked in a molecular layer at a distance of three unit cell thicknesses from the reflecting wall. Fig. 3 shows the temporal variation of the densities of CL-20/MTNP, CL-20, and MTNP crystals at different impact velocities. At 0 ps, the density of the CL-20/MTNP co-crystal was lower than that of CL-20 and higher than that of MTNP, indicating that the addition of MTNP molecules reduced the density of CL-20 crystals. As the shock wavefront propagated, the densities of the three crystals all suddenly *jumped* and then remained stable. However, at an impact velocity of 2 km s^−1^, the peak values of the density jumps were different among the three crystals, decreasing in the order of CL-20 > CL-20/MTNP > MTNP. At an impact velocity of 3 km s^−1^, the densities of CL-20/MTNP and MTNP jumped to approximately 2.75 g cm^−3^, while the maximum compressed density of CL-20 reached 2.9 g cm^−3^. At an impact velocity of 4 km s^−1^, the densities of CL-20/MTNP and MTNP jumped to approximately 2.95 g cm^−3^, while the density of CL-20 reached 3.1 g cm^−3^. The densities of the three crystals reached the peak values of their jumps, then slightly increased, and finally stabilized at 4 ps. At all three impact velocities, the densities of the three crystals decreased in the order of CL-20 > CL-20/MTNP > MTNP at 10 ps.

**Fig. 4:**
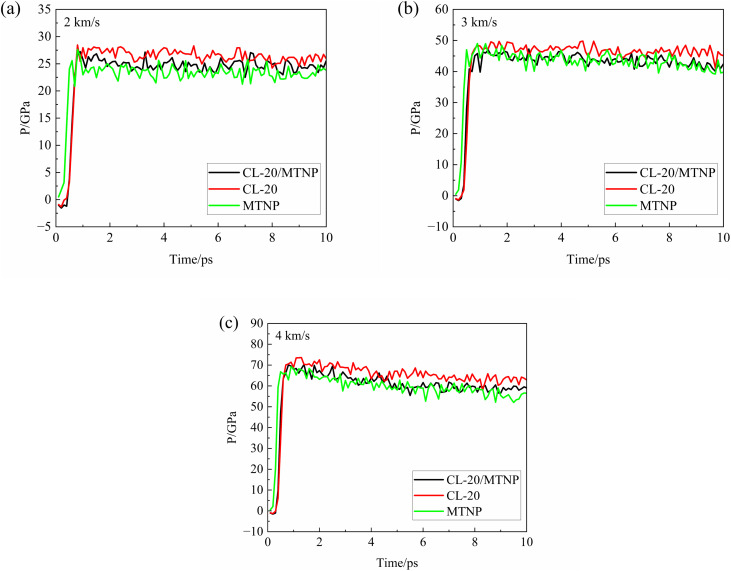
Temporal variation of the pressures of CL-20/MTNP, CL-20, and MTNP crystals at different impact velocities: (a) 2 km s^−1^; (b) 3 km s^−1^; (c) 4 km s^−1^.

The detonation pressure of energetic materials is one of the important parameters for assessing detonation performance. Therefore, the pressure along the impact direction of the molecular layers was calculated during the molecular dynamics simulations. Fig. 4 shows the temporal variation of the pressures of CL-20/MTNP, CL-20, and MTNP crystals at different impact velocities. Variation of the pressure was similar between the CL-20/MTNP co-crystal and the other two single crystals at different impact velocities. The initial pressure of all three crystals was zero. After the shock wavefront passed through, the pressure suddenly jumped and then fluctuated or slightly decreased. The higher the impact velocity, the larger the peak value of the sudden pressure jump, after which the pressure stabilized. Ghule^23^ and Bogdanovaet^24^ obtained two slightly different values for the detonation pressure of CL-20: 44.64 GPa through quantum chemistry calculations and 46.06 GPa from thermodynamic calculations. In contrast, Zhang *et al.*^25^ obtained a slightly higher value of 50.3 GPa for the detonation pressure of CL-20 using the multi-scale shock technique (MSST) and molecular dynamics simulations. In this study, the pressure of the CL-20 crystal wavefront at an impact velocity of 3 km s^−1^ was approximately 48 GPa, which was consistent with the above-mentioned calculation results. This confirmed that our molecular dynamics simulations were reliable and could describe the real propagation of shock waves in explosives at the microscopic level.

**Fig. 5:**
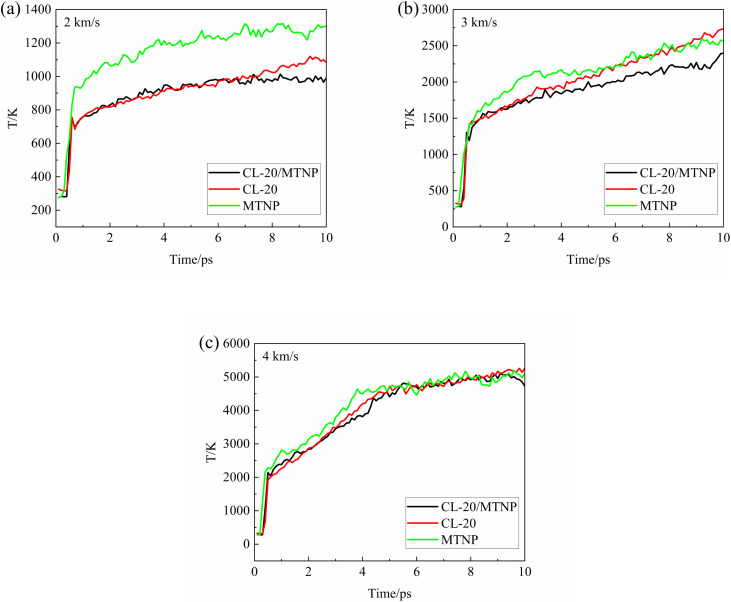
Temporal variation of the temperatures of CL-20/MTNP, CL-20, and MTNP crystals at different impact velocities: (a) 2 km s^−1^; (b) 3 km s^−1^; (c) 4 km s^−1^.

Fig. 5 shows temporal variation of the temperatures of CL-20/MTNP, CL-20, and MTNP crystals at different impact velocities. At these impact velocities, the temperatures of the three crystals followed a general trend after the shock wavefront passed through: an initial sudden jump followed by a slow increase. However, at an impact velocity of 4 km s^−1^, the temperatures of the three crystals stabilized after the slow increase. At all impact velocities, the temperature of the CL-20/MTNP co-crystal decreased for a brief period at approximately 0.6 ps between the sudden jump and slow increase. This indicated that an endothermic reaction occurred during this brief stage. At an impact velocity of 2 km s^−1^, the temperature of MTNP was considerably higher than those of CL-20/MTNP and CL-20 after the shock wavefront passed through. This indicated that the shock wavefront caused severe vibrations of the molecules in the crystals, and the vibrations of CL-20 and MTNP molecules were more severe in the co-crystal than in the corresponding single crystals. At an impact velocity of 3 km s^−1^, the temperatures of the three crystals were similar, although the temperature of MTNP was slightly higher than that of the other crystals in the first 7 ps, and the temperature of CL-20 surpassed that of the other two crystals from 7 ps. At an impact velocity of 4 km s^−1^, both the magnitude and temporal variation of the temperatures of the three crystals were similar, although the temperature of MTNP was slightly higher than that of the other crystals in the first 4 ps.

### 3.3. Chemical species generated in the crystals

**Fig. 6:**
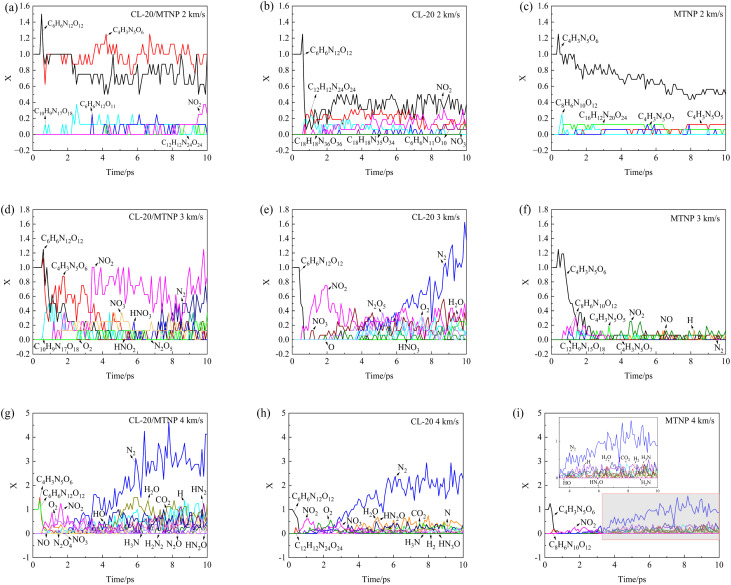
Temporal variation of *X* for CL-20/MTNP, CL-20, and MTNP crystals at different impact velocities. Panels (a–c), (d–f), and (g–i) correspond to impact velocities of 2, 3, and 4 km s^−1^, respectively. Panels (a, d, g), (b, e, h), and (c, f, i) correspond to CL-20/MTNP co-crystal, CL-20, and MTNP, respectively.

Upon impact, nitro-based explosives undergo homolytic cleavage of C–NO_2_ or N–NO_2_ bonds, generating NO_2_, which reacts with other fragments to form N_2_, releasing a massive amount of energy. To compare the generation of products between the co-crystal and single crystals, *X* was defined as the ratio of the number of product species to the number of reactant molecules. Fig. 6 shows the temporal variation of *X* for the main product species in CL-20/MTNP, CL-20, and MTNP crystals at an impact velocity of 2 km s^−1^. In the CL-20/MTNP co-crystal, the number of CL-20 molecules decreased slightly, whereas the number of MTNP molecules did not decrease. However, in the CL-20 and MTNP single crystals, the number of reactant molecules decreased markedly. The main reactions occurring in the CL-20/MTNP co-crystal were the polymerization of CL-20 and MTNP molecules, the polymerization of two CL-20 molecules, the loss of O atoms from CL-20 molecules, and the generation of small NO_2_ molecules. In the CL-20 single crystal, the main reactions were bimolecular (or trimolecular) polymerization and the generation of NO_2_ and NO_3_. In the MTNP single crystal, the main reactions were bimolecular (or tetramolecular) polymerization, the loss of O atoms from MTNP monomers, and the combination of MTNP monomers with free O atoms. Therefore, the presence of MTNP molecules in the co-crystal suppressed the polymerization of CL-20 molecules, prolonged the presence of NO_2_, and delayed the emergence of N_2_ molecules. Similarly, the presence of CL-20 molecules in the co-crystal also suppressed the polymerization of MTNP molecules as well as the decomposition of MTNP molecules into O atoms.

At an impact velocity of 3 km s^−1^, CL-20 and MTNP molecules in the co-crystal disappeared at 4.5 ps and 5.7 ps, respectively. However, CL-20 and MTNP molecules in their corresponding single crystal disappeared at approximately 0.7 ps and 4.2 ps, respectively. This indicated that the decay rates of CL-20 and MTNP molecules were lower in the co-crystal than in the corresponding single crystal. As shown in Fig. 6(d–f), the co-crystal clearly promoted the formation of NO_2_ and NO_3_ and delayed the formation of N_2_.

At an impact velocity of 4 km s^−1^, the reactants in the single crystals and co-crystal disappeared within 1 ps. During this period, the CL-20/MTNP co-crystal generated a variety of small-molecule products, including NO_3_, NO, N_2_O_4_, O_2_, N_2_O, H, HO, HN_2_O, HN_2_, H_3_N, and H_2_N_2_, of which the main small-molecule products were NO_2_, H_2_O, CO_2_, and N_2_. The co-crystal generated these small molecules in greater quantity than the two single crystals. In the CL-20 single crystal, the main small-molecule products were O_2_, NO_3_, HN_2_O, H_2_, H_3_N, and HN_3_O, and in the MTNP single crystal, they were H, HO, HN_2_O, H_2_, H_3_N, and H_2_N. Therefore, the types of products in the co-crystal were more complex than a simple summation of products in the two single crystals.

In summary, the initial chemical reactions of the CL-20/MTNP co-crystal after the passage of the shock wavefront mainly involve the polymerization between CL-20 and MTNP molecules and the formation of NO_2_. The early polymerization process is accompanied by intense molecular configuration distortion, bond angle and bond length deformation, and pre-activation of chemical bonds. These structural rearrangements and the bond breaking associated with NO_2_ formation are endothermic processes that consume local thermal energy, resulting in a temporary decrease in the kinetic temperature. Therefore, as shown in Fig. 5, the temperature of the CL-20/MTNP co-crystal exhibits a brief dip at approximately 0.6 ps.

### 3.4. Chemical bond formation and breakage in the crystals

Chemical reactions entail the formation and breakage of chemical bonds. The number of formation and breakage events of a given type of chemical bond at different times was statistically analyzed. For the convenience of comparison, we define *Y* as the ratio of the difference between bond formation and breakage numbers to the maximum of the formation or breakage number, as expressed in the following equation.^26^ A *Y* value greater than zero indicates net formation of the corresponding type of chemical bond, while a *Y* value less than zero indicates net breakage.
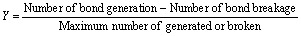


**Fig. 7:**
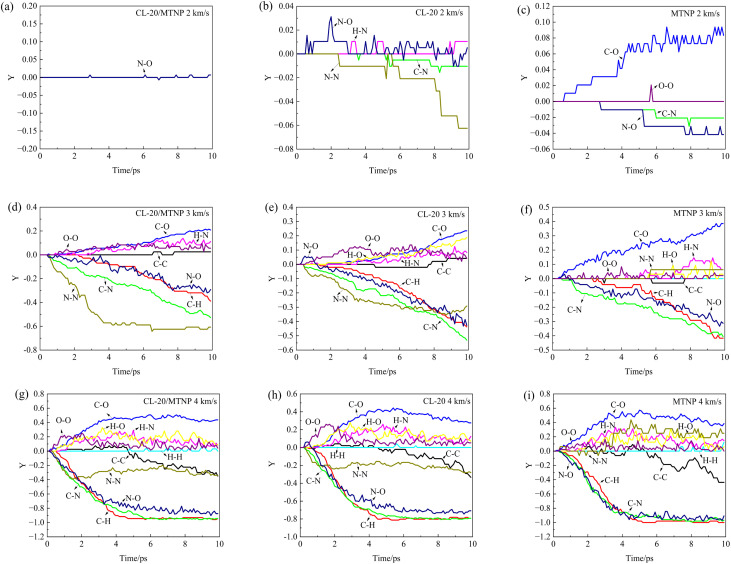
Temporal variation of *Y* for CL-20/MTNP, CL-20, and MTNP crystals at different impact velocities. Panels (a–c), (d–f), and (g–i) correspond to impact velocities of 2, 3, and 4 km s^−1^, respectively. Panels (a, d, g), (b, e, h), and (c, f, i) correspond to CL-20/MTNP co-crystal, CL-20, and MTNP, respectively.

Fig. 7 illustrates the temporal variation of the formation and breakage of different chemical bonds in CL-20/MTNP, CL-20, and MTNP crystals at different impact velocities. At an impact velocity of 2 km s^−1^, chemical bond formation or breakage was negligible in the CL-20/MTNP co-crystal, with minimal transient N–O bond formation and breakage. In the CL-20 single crystal, N–O bond formation occurred first, followed by extensive N–N bond breakage, minor H–N bond formation, and minor C–N bond breakage. In the MTNP single crystal, extensive formation of C–O bonds occurred first, followed by breakage of N–O and C–N bonds and transient formation of a small number of O–O bonds.

The generation of various chemical species in the crystals (section 3.3) can be linked to the above-mentioned formation and breakage of chemical bonds. For example, in the single crystals, polymerization of CL-20 molecules is related to the formation of N–O bonds, while the polymerization of MTNP molecules is related to the formation of C–O bonds. According to the molecular structure of CL-20, the breakage of N–N bonds may generate NO_2_, while the breakage of C–N bonds involves the destruction of its molecular backbone. Similarly, the molecular structure of MTNP indicates that the breakage of N–O bonds may generate free O atoms, and the breakage of C–N bonds may generate NO_2_ or destroy its molecular ring.

At an impact velocity of 3 km s^−1^, N–N bonds in both the co-crystal and CL-20 single crystals exhibited early net breakage, while N–N bonds in the MTNP single crystal showed net formation at approximately 6.0 ps. In the CL-20/MTNP co-crystal, N–O bonds were cleaved, while H–O bonds and other types of chemical bonds, such as C–N, C–H, O–O, and C–O, were unaffected. Moreover, N–N bond breakage occurred less frequently in the co-crystal than in the CL-20 single crystal. At an impact velocity of 4 km s^−1^, changes in the different types of chemical bonds were similar across all three crystals. In the CL-20/MTNP co-crystal, N–O bonds were cleaved, rather than generated. Moreover, O–O, N–N, C–H, C–N, and N–O bonds were changed more frequently in the co-crystal than in the CL-20 single crystal.

### 3.5. Elementary chemical reactions

The chemical reactions of the three crystals were studied in detail. Table 2 lists the earliest and most frequent elementary reactions occurring in the CL-20/MTNP, CL-20, and MTNP crystals at an impact velocity of 2 km s^−1^. The reactions occurring at the other two impact velocities are shown in Tables S1 and S2 (SI). Here, M(i) was defined as molecules outside the study area. At an impact velocity of 2 km s^−1^, the earliest and most frequent reactions occurring in the CL-20/MTNP co-crystal were the polymerization and separation of CL-20 and MTNP molecules. In the CL-20 crystal, the earliest and most frequent chemical reactions were bimolecular (or multimolecular) polymerization and NO_2_ formation, and in the MTNP crystal, the main reaction was polymerization between MTNP molecules. As the impact velocity increased, the molecular weight of the earliest-formed polymer in the three crystals also increased. At high impact velocities, the most frequent elementary reactions mainly occurred between the large number of small-molecule products. At an impact velocity of 3 km s^−1^, the main reactions involved nitrogen oxides, such as NO_2_, and NO_3_, N_2_O_5_. At an impact velocity of 4 km s^−1^, the main reactions occurred between nitrogen and hydrogen compounds, such as N_2_, H, HN, and HN_2_. These patterns were consistent with the results shown in Fig. 6.

**Table 2:** Earliest and most frequent elementary reactions in CL-20/MTNP, CL-20, and MTNP crystals at an impact velocity of 2 km s^−1^

Crystals	Frequencies	Reaction time (ps)	Elementary reactions	Illustration
CL-20/MTNP	6	1.7–5.0	C_4_H_3_N_5_O_6_ → **C**_**4**_**H**_**3**_**N**_**5**_**O**_**6**_**–M(C**_**6**_**H**_**6**_**N**_**12**_**O**_**12**_**)**	First occur
	6	2.0–5.1	C_4_H_3_N_5_O_6_–M(C_6_H_6_N_12_O_12_) → C_4_H_3_N_5_O_6_	
	1	2.6	C_6_H_6_N_12_O_12_ → **C**_**6**_**H**_**6**_**N**_**12**_**O**_**12**_**–M(C**_**4**_**H**_**3**_**N**_**5**_**O**_**6**_**)**	
	3	5.5–5.9	C_4_H_3_N_5_O_6_ → C_4_H_3_N_5_O_6_–M(C_6_H_6_N_11_O_10_)	
	2	5.6–5.8	C_4_H_3_N_5_O_6_–M(C_6_H_6_N_11_O_10_) → C_4_H_3_N_5_O_6_	
	6	2.0–5.1	C_4_H_3_N_5_O_6_–M(**C**_**6**_**H**_**6**_**N**_**12**_**O**_**12**_) → C_4_H_3_N_5_O_6_	Highest frequency
	6	1.7–5.0	C_4_H_3_N_5_O_6_ → **C**_**4**_**H**_**3**_**N**_**5**_**O**_**6**_**–M(C**_**6**_**H**_**6**_**N**_**12**_**O**_**12**_**)**	
	3	5.5–5.9	C_4_H_3_N_5_O_6_ → **C**_**4**_**H**_**3**_**N**_**5**_**O**_**6**_**–M(C**_**6**_**H**_**6**_**N**_**11**_**O**_**10**_**)**	
	2	5.6–5.8	C_4_H_3_N_5_O_6_–M(C_6_H_6_N_11_O_10_) → C_4_H_3_N_5_O_6_	
CL-20	51	0.2–9.9	C_6_H_6_N_12_O_12_ → **C**_**6**_**H**_**6**_**N**_**12**_**O**_**12**_**–M(C**_**6**_**H**_**6**_**N**_**12**_**O**_**12**_**)**	First occur
	7	0.6–8.0	C_6_H_6_N_12_O_12_ → **C**_**6**_**H**_**6**_**N**_**12**_**O**_**12**_**–M(C**_**12**_**H**_**12**_**N**_**24**_**O**_**24**_**)**	
	51	0.7–9.9	C_6_H_6_N_12_O_12_–M(C_6_H_6_N_12_O_12_) → C_6_H_6_N_12_O_12_	
	4	0.7–2.4	C_6_H_6_N_12_O_12_–M(C_6_H_6_N_12_O_12_) → C_6_H_6_N_12_O_12_	
	1	0.8–0.8	C_6_H_6_N_12_O_12_ → **C**_**6**_**H**_**6**_**N**_**12**_**O**_**12**_**–M(C**_**18**_**H**_**18**_**N**_**36**_**O**_**36**_**)**	
	1	0.9–0.9	C_6_H_6_N_12_O_12_–M(C_18_H_18_N_36_O_36_) → C_6_H_6_N_12_O_12_	
	1	1.0–1.0	C_6_H_6_N_12_O_12_ → C_6_H_6_N_12_O_12_–M(**C**_**36**_**H**_**36**_**N**_**72**_**O**_**72**_)	
	1	1.1–1.1	C_6_H_6_N_12_O_12_–M(C_36_H_36_N_72_O_72_) → C_6_H_6_N_12_O_12_	
	51	0.7–9.9	C_6_H_6_N_12_O_12_–M(C_6_H_6_N_12_O_12_) → C_6_H_6_N_12_O_12_	Highest frequency
	51	0.2–9.9	C_6_H_6_N_12_O_12_ → **C**_**6**_**H**_**6**_**N**_**12**_**O**_**12**_**–M(C**_**6**_**H**_**6**_**N**_**12**_**O**_**12**_**)**	
	7	0.6–8.0	C_6_H_6_N_12_O_12_ → **C**_**6**_**H**_**6**_**N**_**12**_**O**_**12**_**–M(C**_**12**_**H**_**12**_**N**_**24**_**O**_**24**_**)**	
	4	0.7–2.4	C_6_H_6_N_12_O_12_–M(C_12_H_12_N_24_O_24_) → C_6_H_6_N_12_O_12_	
	4	5.6–7.1	**C**_**12**_ **H**_**12**_ **N**_**23**_ **O**_**22**_ **→ NO**_**2**_ **+ C**_**12**_ **H**_**12**_ **N**_**22**_ **O**_**20**_	
	4	4.7–9.3	C_6_H_6_N_12_O_12_ → **C**_**6**_**H**_**6**_**N**_**12**_**O**_**12**_**–M(C**_**18**_**H**_**18**_**N**_**35**_**O**_**34**_**)**	
M TNP	2	0.5–0.9	C_4_H_3_N_5_O_6_ + C_4_H_3_N_5_O_6_ → **C**_**8**_**H**_**6**_**N**_**10**_**O**_**12**_	First occur
	8	0.7–7.4	C_4_H_3_N_5_O_6_ → C_4_H_3_N_5_O_6_–M(C_4_H_3_N_5_O_6_)	
	1	1.7	C_4_H_3_N_5_O_6_–M(C_4_H_3_N_5_O_6_) → C_4_H_3_N_5_O_6_–M(C_8_H_6_N_10_O_12_)	
	1	1.8	C_4_H_3_N_5_O_6_–M(C_8_H_6_N_10_O_12_) → C_4_H_3_N_5_O_6_–M(C_4_H_3_N_5_O_6_)	
	8	0.7–7.4	C_4_H_3_N_5_O_6_ → **C**_**4**_**H**_**3**_**N**_**5**_**O**_**6**_**–M(C**_**4**_**H**_**3**_**N**_**5**_**O**_**6**_**)**	Highest frequency
	2	0.5–0.9	C_4_H_3_N_5_O_6_ +C_4_H_3_N_5_O_6_ → **C**_**8**_**H**_**6**_**N**_**10**_**O**_**12**_	
	2	3.9–4.3	C_16_H_12_N_20_O_24_–M(C_8_H_6_N_10_O_12_) → **C**_**12**_**H**_**9**_**N**_**15**_**O**_**18**_**–M(C**_**4**_**H**_**3**_**N**_**5**_**O**_**6**_**)** + **C**_**4**_**H**_**3**_**N**_**5**_**O**_**6**_**–M(C**_**4**_**H**_**3**_**N**_**5**_**O**_**6**_**)**	

### 3.6. Initial chemical reactions

**Fig. 8:**
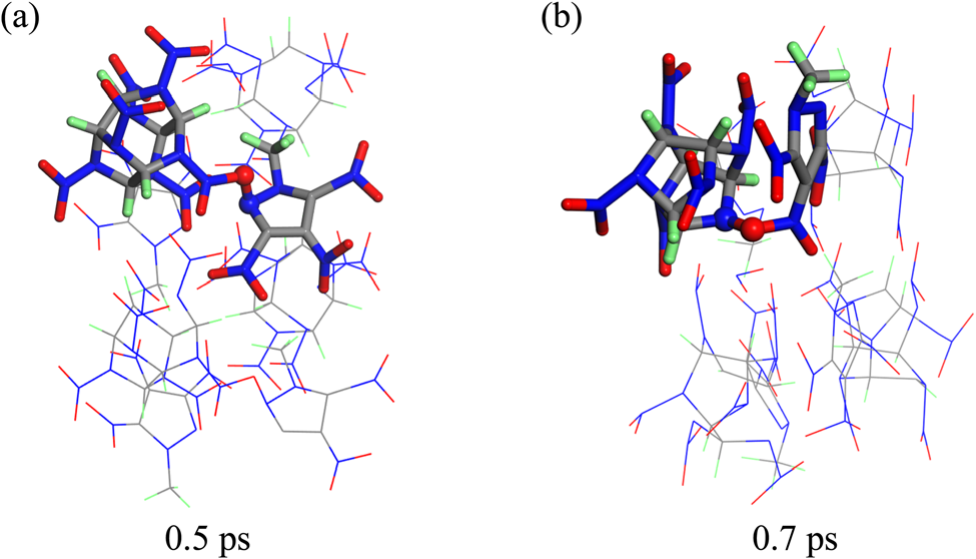
Molecular snapshots of the CL-20/MTNP co-crystal at an impact velocity of 3 km s^−1^. (a) corresponds to 0.5 ps, and (b) corresponds to 0.7 ps.

To investigate the initial chemical reaction triggered by the shock wavefront, we conducted a tracking analysis of the molecular structures. There are two possible paths for the formation of the N–O bond between CL-20 and MTNP molecules. First, the O atom of the NO_2_ group of the CL-20 molecule forms a bond with an isolated N atom in the MTNP molecular framework, as shown in Fig. 8(a). Second, the O atom of the NO_2_ group of the MTNP molecule forms a chemical bond with the N atom of the five-membered ring of the CL-20 molecule, which is directly bonded to the NO_2_ group, as illustrated in Fig. 8(b). The former may not lead to cleavage of the N–NO_2_ bond in CL-20, whereas the latter will directly lead to the breakage of the N–NO_2_ bond in the CL-20 molecule.

**Fig. 9:**
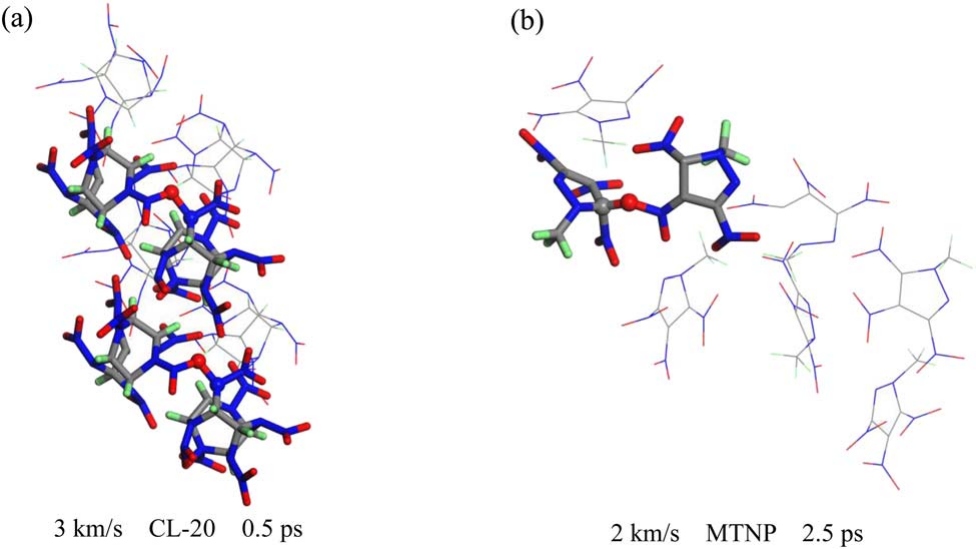
Molecular snapshots of CL-20 (a) and MTNP (b) crystals at different impact velocities.

In the CL-20 single crystal, bimolecular polymerization is primarily achieved through the formation of N–O bonds. The O atom of the NO_2_ group of a CL-20 molecule combines with the N atom of the NO_2_ group of another CL-20 molecule, as shown in Fig. 9(a). This proposed reaction aligns with our previous research on the initial chemical reaction of CL-20 under impact.^27^ In the MTNP single crystal, bimolecular polymerization is primarily achieved through the formation of C–O bonds, as illustrated in Fig. 9(b). The O atom of the NO_2_ group of an MTNP molecule forms a chemical bond with the C atom of the ring of another MTNP molecule.

## 4. Conclusions

In this study, the propagation of shock waves in the CL-20/MTNP co-crystal and the corresponding single crystals at different impact velocities was simulated using the ReaxFF-lg reactive force field and molecular dynamics. Moreover, changes in thermodynamic properties and initiated chemical reactions were analyzed using in-house programs, and the reasons for the reduced sensitivity of the CL-20/MTNP co-crystal were explored. After the shock wavefront propagated through the crystals, the compressed density of the CL-20/MTNP co-crystal was significantly lower than that of the CL-20 single crystal, whereas the pressure and temperature of the two types of crystals were not significantly different. Therefore, the detonation performance of the CL-20/MTNP co-crystal is likely similar to that of the CL-20 single crystal. Polymerization between CL-20 and MTNP molecules in the co-crystal occurred through the formation of N–O bonds, initially generating the main small-molecule product of NO_2_ and subsequently generating the intermediate products of NO_3_, HNO_2_, HNO_3_, N_2_O_5_, and O_2_. Moreover, collisions between CL-20 molecules occurred less frequently in the CL-20/MTNP co-crystal than in the CL-20 single crystal, indirectly suppressing the generation of NO_2_ from CL-20 molecules. Moreover, because the decomposition of MTNP molecules required heat absorption, the generation of intermediate products from CL-20 molecules was prolonged. This may be the reason for the reduced sensitivity of the CL-20/MTNP co-crystal. Furthermore, differences between the co-crystal and single crystals were only evident at medium and low impact velocities.

## Author contributions

Conceptualization, formal analysis, methodology and software, F.W.; investigation, project administration and resources, G.D.; data curation, H.Z., L.Y., W.B. and Y.L.; writing—original draft and writing—review and editing, Y.L., W.H., S.W. and D.Z. All authors have read and agreed to the published version of the manuscript.

## Conflicts of interest

The authors declare no conflict of interest regarding the publication of this paper.

## Supplementary Material

RA-016-D6RA01177B-s001

## Data Availability

All data supporting the findings of this study are included within the article and its supplementary information (SI). Supplementary information: the earliest and most frequent elementary reactions occurring in CL-20/MTNP, CL-20, and MTNP crystals at an impact velocity of 3 km s^−1^ (Table S1) and 4 km s^−1^ (Table S2). See DOI: https://doi.org/10.1039/d6ra01177b.
