# Supplementary material for: Mechanism of the reduced shock sensitivity of CL-20/MTNP co-crystals from reactive molecular dynamics simulations
Source: RSC Adv. 2026 Apr 13;16(21):19187–97. doi: 10.1039/d6ra01177b (PMC13071387; doi:10.1039/d6ra01177b)
Supplement: RA-016-D6RA01177B-s001 [file RA-016-D6RA01177B-s001.pdf]

Supplementary Information (SI) for RSC Advances.
This journal is © The Royal Society of Chemistry 2026

Mechanism of the reduced shock sensitivity of CL-20/MTNP co-crystals from reactive molecular dynamics simulations

Fuping Wang, Guangyan Du, Huihan Zhang, Liufei Yang, Wanru Bi, Yiyang Li, Yaowen Liu, Wenfang Hou, Songen Wang, Dongqiang Zhang*

School of Chemistry and Material Science, Langfang Normal University, Langfang, 065000, China

*Correspondence: wangfuping@lfnu.edu.cn

Supplementary Materials:

The earliest and most frequent elementary reactions occurring in CL-20/MTNP, CL-20, and MTNP crystals at an impact velocity of 3 km/s (Table S1) and 4 km/s (Table S2).

Table S1 Earliest and most frequent elementary reactions in CL-20/MTNP, CL-20, and MTNP crystals at an impact velocity of 3 km/s

Crystals	Frequencies	Reaction time(ps)	Elementary reactions	Illustration
CL-20/MTNP	3	0.2-0.2	$C_4H_3N_5O_6 \rightarrow \mathbf{C_4H_3N_5O_6\text{-}M(C_6H_6N_{12}O_{12})}$	First occur
	6	0.3-0.3	$C_6H_6N_{12}O_{12} \rightarrow C_6H_6N_{12}O_{12}\text{-}M(C_{10}H_9N_{17}O_{18})$	
	2	0.3-0.3	$C_4H_3N_5O_6\text{-}M(C_6H_6N_{12}O_{12})+C_6H_6N_{12}O_{12} \rightarrow \mathbf{C_{10}H_9N_{17}O_{18}\text{-}M(C_6H_6N_{12}O_{12})}$	
	1	0.3-0.3	$C_4H_3N_5O_6+C_6H_6N_{12}O_{12}\text{-}M(C_{10}H_9N_{17}O_{18}) \rightarrow C_{10}H_9N_{17}O_{18}\text{-}M(C_{34}H_{30}N_{56}O_{60})$	
	1	0.3-0.3	$C_4H_3N_5O_6+C_6H_6N_{12}O_{12}+C_6H_6N_{12}O_{12}\text{-}M(C_{10}H_9N_{17}O_{18}) \rightarrow \mathbf{C_{16}H_{15}N_{29}O_{30}\text{-}M(C_{20}H_{18}N_{33}O_{33})}$	
	1	0.4-0.4	$C_{10}H_9N_{17}O_{18}\text{-}M(C_6H_6N_{12}O_{12}) \rightarrow C_{10}H_9N_{17}O_{18}\text{-}M(C_{14}H_{12}N_{22}O_{24})$	
	3	0.4-0.4	$C_6H_6N_{12}O_{12} \rightarrow \mathbf{C_6H_6N_{12}O_{12}\text{-}M(C_4H_3N_5O_6)}$	
	1	0.4-0.4	$C_{16}H_{15}N_{29}O_{30}\text{-}M(C_{20}H_{18}N_{33}O_{33}) \rightarrow C_{10}H_9N_{17}O_{18}+C_6H_6N_{12}O_{12}$	
	6	0.4-1.6	$\mathbf{C_6H_6N_{12}O_{12} \rightarrow C_6H_6N_{12}O_{12}\text{-}M(C_{10}H_9N_{17}O_{18})}$	Highest frequency
	6	1.4-6.3	$\mathbf{NO_2+NO_2 \rightarrow N_2O_4}$	
	4	5.0-7.6	$\mathbf{N_2O_5 \rightarrow NO_2+NO_3}$	
	3	0.9-2.3	$C_6H_6N_{12}O_{12}\text{-}M(C_{10}H_9N_{17}O_{18}) \rightarrow C_6H_6N_{12}O_{12}$	
	3	0.8-1.4	$C_6H_6N_{12}O_{12}\text{-}M(C_4H_3N_5O_6) \rightarrow C_6H_6N_{12}O_{12}$	
	3	0.6-1.3	$C_6H_6N_{12}O_{12} \rightarrow \mathbf{C_6H_6N_{12}O_{12}\text{-}M(C_4H_3N_5O_6)}$	
	3	0.3-1.2	$C_4H_3N_5O_6 \rightarrow \mathbf{C_4H_3N_5O_6\text{-}M(C_6H_6N_{12}O_{12})}$	
	3	1.9-5.0	$\mathbf{N_2O_4 \rightarrow NO_2+NO_2}$	
CL-20	1	0.2-0.2	$C_6H_6N_{12}O_{12} \rightarrow \mathbf{C_6H_6N_{12}O_{12}\text{-}M(C_6H_6N_{12}O_{12})}$	First occur
	1	0.3-0.3	$C_6H_6N_{12}O_{12}+C_6H_6N_{12}O_{12} \rightarrow \mathbf{C_{12}H_{12}N_{24}O_{24}}\text{-}M(C_{48}H_{49}N_{96}O_{91})$	
	1	0.3-0.3	$C_6H_6N_{12}O_{12}+C_6H_6N_{12}O_{12}+C_6H_6N_{12}O_{12} \rightarrow \mathbf{C_{18}H_{18}N_{36}O_{36}}\text{-}M(C_{30}H_{29}N_{59}O_{62})$	

	1	0.3-0.3	$C_6H_6N_{12}O_{12}+C_6H_6N_{12}O_{12}+C_6H_6N_{12}O_{12}+C_6H_6N_{12}O_{12}\text{-M}(C_6H_6N_{12}O_{12})\rightarrow C_{24}H_{24}N_{48}O_{48}\text{-M}(C_{24}H_{23}N_{43}O_{43})$	
	1	0.4-0.4	$C_{24}H_{24}N_{48}O_{48}\text{-M}(C_{24}H_{23}N_{43}O_{43})\rightarrow C_{18}H_{18}N_{36}O_{36}\text{-M}(C_{36}H_{35}N_{66}O_{64})+C_6H_6N_{12}O_{12}\text{-M}(C_6H_6N_{12}O_{12})$	
	1	0.4-0.4	$C_{18}H_{18}N_{36}O_{36}\text{-M}(C_{30}H_{29}N_{59}O_{62})\rightarrow C_{12}H_{12}N_{24}O_{24}\text{-M}(C_{42}H_{40}N_{81}O_{79})+C_6H_6N_{12}O_{12}\text{-M}(C_6H_6N_{12}O_{12})$	
	1	0.4-0.4	$C_{18}H_{18}N_{36}O_{36}\text{-M}(C_{30}H_{29}N_{56}O_{56})\rightarrow \mathbf{C_{12}H_{12}N_{24}O_{24}\text{-M}(C_{54}H_{53}N_{101}O_{98})}+C_6H_6N_{12}O_{12}\text{-M}(C_6H_6N_{12}O_{12})$	
	1	0.4-0.4	$C_{12}H_{12}N_{24}O_{24}\text{-M}(C_{48}H_{49}N_{96}O_{91})\rightarrow C_6H_6N_{12}O_{12}\text{-M}(C_{12}H_{12}N_{24}O_{24})+C_6H_6N_{12}O_{12}\text{-M}(C_6H_6N_{12}O_{12})$	
	4	5.8-8.1	$\mathbf{NO_2\text{-M}(NO_3)+N_2}\rightarrow N_3O_2\text{-M}(NO_3)$	Highest frequency
	3	5.9-8.2	$N_3O_2\text{-M}(NO_3)\rightarrow NO_2\text{-M}(NO_3)+N_2$	
	2	8.1-9.2	$HO\rightarrow HO\text{-M}(NO_2)$	
	2	7.5-8.1	$\mathbf{N\text{-M}(HN_2O)}\rightarrow N\text{-M}(N_2)$	
	2	7.4-7.6	$N\text{-M}(N_2)\rightarrow N\text{-M}(HN_2O)$	
	2	7.3-7.6	$\mathbf{NO_2\text{-M}(O)}+N_2\rightarrow N_3O_2\text{-M}(O)$	
	2	6.2-8.6	$HO\rightarrow HO\text{-M}(N_2)$	
	2	5.8-6.1	$\mathbf{O\text{-M}(O)+NO}\rightarrow NO_2\text{-M}(O)$	
	2	5.7-5.9	$NO_2\text{-M}(O)\rightarrow O\text{-M}(O)+NO$	
	2	1.6-2.2	$\mathbf{NO_2\text{-M}(NO_2)}\rightarrow NO_2\text{-M}(N_2O_4)$	
	2	1.6-1.9	$NO_2\rightarrow NO_2\text{-M}(NO_2)$	
	2	1.5-2.1	$\mathbf{NO_2\text{-M}(N_2O_4)}\rightarrow NO_2\text{-M}(NO_2)$	
MTNP	6	0.3-0.5	$C_4H_3N_5O_6\rightarrow \mathbf{C_4H_3N_5O_6\text{-M}(C_4H_3N_5O_6)}$	First occur
	2	0.4-0.6	$C_4H_3N_5O_6\text{-M}(C_4H_3N_5O_6)\rightarrow C_4H_3N_5O_6$	
	1	0.4	$C_4H_3N_5O_6+C_4H_3N_5O_6\text{-M}(C_4H_3N_5O_6)\rightarrow \mathbf{C_8H_6N_{10}O_{12}\text{-M}(C_8H_6N_{10}O_{12})}$	
	1	0.4	$C_4H_3N_5O_6+C_4H_3N_5O_6\text{-M}(C_4H_3N_5O_6)\rightarrow C_8H_6N_{10}O_{12}\text{-M}(C_4H_3N_5O_6)$	
	1	0.4	$C_4H_3N_5O_6+C_4H_3N_5O_6\rightarrow C_8H_6N_{10}O_{12}\text{-M}(\mathbf{C_{55}H_{37}N_{62}O_{74}})$	
	1	0.4	$C_4H_3N_5O_6+C_4H_3N_5O_6\rightarrow C_8H_6N_{10}O_{12}$	
	6	0.3-0.5	$C_4H_3N_5O_6\rightarrow \mathbf{C_4H_3N_5O_6\text{-M}(C_4H_3N_5O_6)}$	Highest frequency
	3	4.4-5.5	$NO_2\rightarrow NO_2\text{-M}(C_4H_3N_5O_6)$	
	2	0.9-1.1	$C_7H_3N_{10}O_{12}\rightarrow C_3N_5O_7+C_4H_3N_5O_5$	
	2	7.1-9.0	$\mathbf{NO_2\rightarrow NO_2\text{-M}(NO_2)}$	
	2	0.4-0.6	$C_4H_3N_5O_6\text{-M}(C_4H_3N_5O_6)\rightarrow C_4H_3N_5O_6$	

Table S2 Earliest and most frequent elementary reactions in CL-20/MTNP, CL-20, and MTNP crystals at an impact velocity of 4 km/s

Crystals	Frequencies	Reaction time(ps)	Elementary reactions	Illustration
CL-20/ MTNP	1	0.2-0.2	$\mathrm{C_4H_3N_5O_6+C_4H_3N_5O_6+C_6H_6N_{12}O_{12}+C_6H_6N_{12}O_{12}+C_6H_6N_{12}O_{12}+C_6H_6N_{12}O_{12}}\rightarrow\mathbf{C_{32}H_{30}N_{58}O_{60}-M(C_{142}H_{130}N_{242}O_{251})}$	First occur
	1	0.3-0.3	$\mathrm{C_{32}H_{30}N_{58}O_{60}-M(C_{142}H_{130}N_{242}O_{251})+C_6H_6N_{12}O_{12}+C_6H_6N_{12}O_{12}+C_6H_6N_{12}O_{12}+C_6H_6N_{12}O_{12}}\rightarrow\mathrm{C_{12}H_{12}N_{24}O_{24}-M(C_{10}H_9N_{17}O_{18})+C_{12}H_{12}N_{24}O_{24}-M(C_{14}H_{12}N_{22}O_{23})+C_{16}H_{15}N_{29}O_{30}-M(C_{117}H_{107}N_{193}O_{191})+C_{16}H_{15}N_{29}O_{30}-M(C_6H_6N_{12}O_{11})}$	
	1	0.4-0.4	$\mathrm{C_{16}H_{15}N_{29}O_{30}-M(C_6H_6N_{12}O_{11})}\rightarrow\mathrm{NO_2+C_{10}H_9N_{16}O_{17}-M(C_{10}H_9N_{17}O_{20})+C_6H_6N_{12}O_{11}}$	
	1	0.4-0.4	$\mathrm{C_{16}H_{15}N_{29}O_{30}-M(C_{117}H_{107}N_{193}O_{191})}\rightarrow\mathrm{O-M(C_4H_3N_5O_6)+CH_3N_2O+C_{15}H_{12}N_{27}O_{28}-M(C_{137}H_{121}N_{224}O_{218})}$	
	1	0.4-0.4	$\mathrm{C_{12}H_{12}N_{24}O_{24}-M(C_{10}H_9N_{17}O_{18})+C_{12}H_{12}N_{24}O_{24}-M(C_{14}H_{12}N_{22}O_{23})+C_4H_3N_5O_6}\rightarrow\mathbf{O}\mathrm{-M(C_{20}H_{18}N_{34}O_{35})}+\mathbf{2O_2}+\mathbf{NO_2}+\mathrm{C_{16}H_{15}N_{28}O_{25}-M(C_4H_3N_5O_6)}+\mathbf{C_6H_6N_{12}O_{11}}+\mathrm{C_6H_6N_{12}O_{11}-M(C_4H_3N_5O_6)}$	
	13	4.3-9.8	$\mathbf{N_2-M(H)}\rightarrow\mathrm{N_2}$	Highest frequency
	13	4.2-9.2	$\mathrm{N_2}\rightarrow\mathrm{N_2-M(H)}$	
	11	5.1-9.8	$\mathrm{N_2}\rightarrow\mathrm{N_2-M(HO)}$	
	11	5.0-9.7	$\mathbf{N-M(N)}\rightarrow\mathrm{N-M(HN)}$	
	10	3.1-9.9	$\mathrm{N_2-M(HO)}\rightarrow\mathrm{N_2}$	
	8	5.1-9.4	$\mathbf{O-M(H_2)}\rightarrow\mathrm{O-M(H_2)}$	
	8	4.0-9.8	$\mathrm{O-M(H_2)}\rightarrow\mathrm{O-M(H)}$	
CL-20	1	0.2-0.2	$\mathrm{C_6H_6N_{12}O_{12}+C_6H_6N_{12}O_{12}}\rightarrow\mathbf{C_{12}H_{12}N_{24}O_{24}}$	First occur
	7	0.2-0.3	$\mathrm{C_6H_6N_{12}O_{12}}\rightarrow\mathrm{C_6H_6N_{12}O_{12}-M(C_6H_6N_{12}O_{12})}$	
	1	0.3-0.3	$\mathrm{C_6H_6N_{12}O_{12}}\rightarrow\mathbf{C_6H_6N_{12}O_{12}-M(C_{12}H_{12}N_{24}O_{24})}$	
	1	0.3-0.3	$\mathrm{C_{12}H_{12}N_{24}O_{24}+C_6H_6N_{12}O_{12}-M(C_6H_6N_{12}O_{12})+C_6H_6N_{12}O_{12}-M(C_6H_6N_{12}O_{12})+C_6H_6N_{12}O_{12}-M(C_6H_6N_{12}O_{12})}\rightarrow\mathrm{O-M(O)}+\mathbf{NO_2}+\mathbf{C_{30}H_{30}N_{59}O_{57}(C_6H_6N_{12}O_{12}+C_6H_6N_{12}O_{12}+C_{18}H_{18}N_{35}O_{33})}\mathrm{-M(C_{176}H_{174}N_{330}O_{308})}$	
	1	0.4-0.4	$\mathrm{C_6H_6N_{12}O_{12}}\rightarrow\mathbf{NO_2}+\mathrm{C_6H_6N_{11}O_{10}}$	
	1	0.4-0.4	$\mathrm{C_{30}H_{30}N_{59}O_{57}}\mathbf{(C_6H_6N_{12}O_{12}+C_6H_6N_{12}O_{12}+C_{18}H_{18}N_{35}O_{33})}\mathrm{-M(C_{176}H_{174}N_{330}O_{308})+C_6H_6N_{12}O_{12}+C_6H_6N_{12}O_{12}-M(C_{12}H_{12}N_{24}O_{24})+C_6H_6N_{12}O_{12}-M(C_6H_6N_{12}O_{12})+C_6H_6N_{12}O_{12}-M(C_6H_6N_{12}O_{12})+C_6H_6N_{12}O_{12}-M(C_6H_6N_{12}O_{12})+C_6H_6N_{12}O_{12}-M(C_6H_6N_{12}O_{12})}\rightarrow\mathbf{4NO_2}+\mathrm{C_{18}H_{18}N_{33}O_{30}-M(C_{12}H_{12}N_{25}O_{25})+C_{48}H_{48}N_{94}O_{91}-M(C_{252}H_{242}N_{478}O_{449})}$	
	1	0.4-0.4	$\mathrm{O-M(O)}\rightarrow\mathrm{O-M(C_6H_8N_{11}O_9)}$	
	19	2.1-9.9	$\mathrm{N_2}\rightarrow\mathbf{N_2-M(H)}$	Highest frequency
	18	2.2-9.1	$\mathrm{N_2-M(H)}\rightarrow\mathrm{N_2}$	
	14	3-9.9	$\mathrm{N-M(N)}\rightarrow\mathrm{N-M(HN)}$	
	13	2.8-9.7	$\mathbf{N_2-M(HO)}\rightarrow\mathrm{N_2}$	
	11	2.7-9.6	$\mathrm{N_2}\rightarrow\mathrm{N_2-M(HO)}$	

	11	2.7-9.5	**N-M(HN)**→N-M(N)	
	10	2.7-9.4	O-M(H_2)→O-M(H)	
MTNP	3	0.2-0.3	$C_4H_3N_5O_6$→**$C_4H_3N_5O_6$-M($C_4H_3N_5O_6$)**	First occur
	1	0.2-0.2	$C_4H_3N_5O_6$→$C_4H_3N_5O_6$-M($C_7H_4N_9O_{10}$)	
	1	0.2-0.2	$C_4H_3N_5O_6$+$C_4H_3N_5O_6$→$C_8H_6N_{10}O_{12}$-M($C_7H_5N_8O_9$)	
	1	0.2-0.2	$C_4H_3N_5O_6$+$C_4H_3N_5O_6$→$C_8H_6N_{10}O_{12}$-M($C_{65}H_{43}N_{75}O_{97}$)	
	1	0.3-0.3	$C_4H_3N_5O_6$-M($C_7H_4N_9O_{10}$)→$C_4H_3N_5O_6$	
	1	0.3-0.3	$C_4H_3N_5O_6$+$C_4H_3N_5O_6$+$C_4H_3N_5O_6$+$C_4H_3N_5O_6$+$C_4H_3N_5O_6$+$C_4H_3N_5O_6$+ $C_4H_3N_5O_6$+$C_4H_3N_5O_6$-M($C_4H_3N_5O_6$)+$C_4H_3N_5O_6$-M($C_4H_3N_5O_6$)+C_8H_6 $N_{10}O_{12}$-M($C_{65}H_{43}N_{75}O_{97}$)+$C_8H_6N_{10}O_{12}$-M($C_7H_5N_8O_9$)→$C_{16}H_{12}N_{20}O_{24}$-M($C_{23}H_{16}N_{27}O_{30}$)+$C_{36}H_{27}N_{45}O_{54}$-M($C_{104}H_{78}N_{125}O_{155}$)	
	1	0.4-0.4	$C_6H_6N_{12}O_{12}$→$C_6H_6N_{12}O_{12}$-M($C_4H_3N_5O_6$)	
	13	3.7-9.7	**N-M(HN)**→N-M(N)	Highest frequency
	11	3.6-9.7	N-M(N)→N-M(HN)	
	10	2.1-9.1	**N_2-M(H)**→N_2	
	8	2.3-8.6	**O-M(H_2)**→O-M(H)	
	8	2.4-9.7	N_2→N_2-M(H)	
	6	3.6-9.9	N_2-M(HO)→N_2	
	6	4.0-8.8	O-M(H)→O-M(H_2)	
